# Cyclomatic Complexity: A Novel Approach for Analyzing Movement with Machine Learning to Predict Mild Cognitive Impairment

**DOI:** 10.1002/alz.090391

**Published:** 2025-01-09

**Authors:** Brian B Johnson, Britni A Bolstad

**Affiliations:** ^1^ University of Minnesota, Minneapolis, MN USA; ^2^ WellBe Senior Medical, Atlanta, GA USA

## Abstract

**Background:**

Patterns of human movement evolve as an individual experiences the progression from cognitively intact (CIN) to mild cognitive impairment (MCI) and finally dementia and Alzheimer’s Disease (AD). Quantification of movement with the goal of identifying MCI though step counts alone do not take into consideration the floor layout of a home. Cyclomatic complexity is an approach to normalizing differences in the individual’s living space to better predict the transition from CIN to MCI. This approach could be especially beneficial for organizations that manage large populations of individuals that prefer to age‐in‐place and hospital‐at‐home environments.

**Method:**

Cyclomatic complexity is the sequence of movements through rooms rather than room transitions or step counts alone. For example, if two rooms are in a straight line (north to south) this has less cyclomatic complexity than two rooms that are diagonal (north to southwest). Cyclomatic complexity is represented as M = E − N + 2P where E is the amount of walking paths, N is the number of rooms, P is the total number of connected rooms (Khan & Jacobs). Movement entropy considers the decrease in the sequence rather than for separate rooms. Monitoring these variables with ambient sensors could provide groundbreaking insights on the quality of life of individuals as they age.

**Result:**

Early studies have shown cyclomatic complexity to be a statically significant predictor of the progression of CI to MCI six months before diagnosis according to “Prediction of Mild Cognitive Impairment Using Movement Complexity” by Dr. Taha Khan and Dr. Peter G. Jacobs.

**Conclusion:**

An estimation of movement to correlate the progression of MCI would benefit from using both sensor data on total step count in addition to cyclomatic complexity. Room transitions alone are unable to produce *p* < 0.05. Cyclomatic complexity adds potentially higher predictive value for MCI because it measures sequences of movement instead of quantity which varies depending on the individual’s home. Additional research is required to understand the technology requirements and data science to use cyclomatic complexity in the standard of care.

